# Development and evaluation of a hybrid capture-based NGS panel for comprehensive detection of respiratory pathogens

**DOI:** 10.1038/s41598-025-26421-2

**Published:** 2025-11-26

**Authors:** Jeong-Ah Kim, Jeong-Min Kim, Chaeyoung Lee, Il-Hwan Kim, Daehwan Lee, Heesoo Lee, Jaehwan Jeong, Eun-Jin Kim

**Affiliations:** 1 Division of Emerging Infectious Diseases, Department of Laboratory Diagnosis and Analysis, Korea Disease Control and Prevention Agency (KDCA), Osong Health Technology Administration Complex, 187, Osongsaengmyeong 2-ro, Osong-eup, Heungdeok-gu, Cheongju-si, Chungcheongbuk-do Republic of Korea; 2 R&D Team, Celemics, Inc., Seoul, Republic of Korea

**Keywords:** Next-generation sequencing (NGS), Respiratory pathogens, Infectious diseases, Biological techniques, Computational biology and bioinformatics, Diseases, Microbiology

## Abstract

Infectious diseases caused by diverse pathogens—including viruses, bacteria, and fungi—pose a significant diagnostic challenge, especially when conventional assays fail to identify the causative agents. Next-generation sequencing (NGS), particularly hybrid-capture-based NGS, offers a culture-independent approach for comprehensive pathogen detection directly from clinical samples. In this study, we developed a hybrid-capture-based NGS panel targeting 85 respiratory pathogens (62 viruses, 23 bacteria/fungi) and evaluated its analytical and clinical performance. Analytical validation using 50 pathogens demonstrated high sensitivity, with 10× genome coverage ≥ 96% in 32 of 36 viral targets and ≥ 95% in all 14 bacterial/fungal targets. The panel maintained ≥ 80% genome coverage at Ct values up to 34. Clinical assessment of 561 respiratory specimens identified 25 pathogen species, achieving ≥ 90% genome coverage in 57.5% of virus-positive and 83.6% of bacteria-positive samples. Notably, additional pathogens were detected in high Ct value undetected by RT-PCR, and 35 cases of viral–viral and viral–bacterial co-infections were identified. This approach improves the detection performance for respiratory infections of unknown etiology and serves as a practical platform for the detection of emerging pathogens and public health preparedness. This technology has significant potential for applications in infectious disease surveillance and development of vaccines and therapeutics.

**Supplementary Information:**

The online version contains supplementary material available at 10.1038/s41598-025-26421-2.

## Introduction

Infectious diseases remain a persistent global public health threat, with etiological agents spanning a wide spectrum, including bacteria, viruses, fungi, and parasites. In particular, infections caused by unknown pathogens that are not detectable by conventional molecular diagnostics or culture-based methods can lead to delayed diagnosis and treatment, resulting in increased patient mortality and significant socioeconomic impact due to uncontrolled transmission^1,2^. The emergence of novel pathogens, such as SARS-CoV, MERS-CoV, and SARS-CoV-2, underscores the critical risk posed by undiagnosed infections and highlights the need for early detection and rapid response systems^3^.

Against this backdrop, next-generation sequencing (NGS) has gained attention as a promising tool for diagnosing infectious diseases. Among various NGS approaches, hybrid-capture-based NGS has emerged as a powerful method capable of directly analyzing pathogen genomes from clinical specimens without the need for culture or PCR amplification^4,5^. This approach enables not only the precise identification of pathogens but also genomic characterization, variant tracking, and vaccine target discovery^6^. Hybrid-capture operates by designing probes based on reference sequences of target pathogens, allowing for the selective enrichment of specific genomes. When broad probe panels incorporating multiple reference sequences are used, this method can capture not only individual pathogens, but also a wide range of genomic variants and even previously uncharacterized pathogens with homologous sequences^7^.

Several hybrid-capture-based NGS panels are now commercially available, including SureSelect Target Enrichment (Agilent), DNA Prep with Enrichment (Illumina), KAPA Target Enrichment (Roche), QIAseq xHYB (QIAGEN), and a Respiratory Virus Research Panel (Twist Bioscience)^8,9^. However, many of these panels are limited to specific pathogen groups, typically viruses or bacteria, restricting their capacity to detect co-infections or to simultaneously detect diverse pathogen groups, such as viruses, bacteria, and fungi, that may cause respiratory infections.

In countries such as the United States and Germany, syndromic surveillance systems enable the early detection of infectious disease outbreaks^10,11^. The Korea Disease Control and Prevention Agency (KDCA) is pursuing a diagnostic framework capable of identifying unknown pathogens to prepare for future emerging infectious diseases and pandemics^12^. In the present study, we developed a hybrid-capture-based NGS panel capable of simultaneously detecting and analyzing viruses, bacteria, and fungi that may cause respiratory syndromes. We also established a bioinformatics analysis pipeline and validated the performance of the panel using both clinical specimens and reference materials. This platform is expected to enhance the early detection of novel pathogens and facilitate rapid diagnostic development. The application of this technology may contribute meaningfully to public health preparedness, including pathogen characterization and the development of vaccines and therapeutics^13^.

## Results

### The respiratory panel and in silico performance

A total of 85 respiratory pathogens (62 viruses and 23 bacterial and fungal species) were selected based on clinical relevance and prevalence (Supplementary Table 1). For viral pathogens, probes were designed to capture the whole genome, whereas for bacterial and fungal pathogens, probes targeted specific loci that enable species-level discrimination (Supplementary Table 2). To assess probe specificity, in silico simulations were performed using reference genomes. Simulated NGS reads were generated assuming a 10% variant frequency to mimic natural sequence diversity. The analysis revealed no cross-reactivity among the reference genomes of the included pathogens (Supplementary Tables 3 and 4), confirming probe specificity.

### Test of hybrid capture-based sequencing in positive samples

**Fig. 1:**
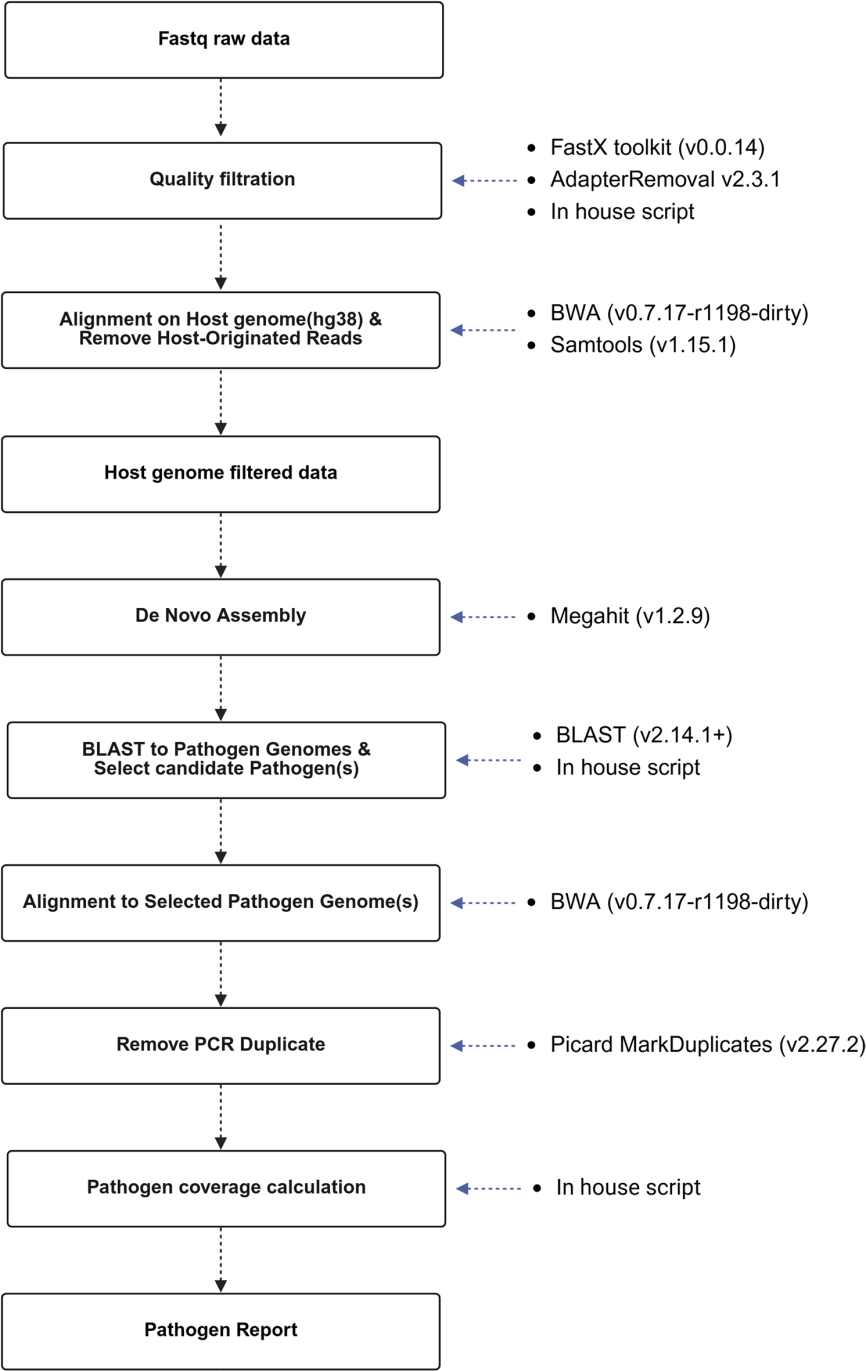
Schematic of the NGS data analysis pipeline for respiratory syndrome. This figure illustrates a bioinformatics pipeline and its components, which are typically used to process NGS data. Created using Bio-render.

**Fig. 2:**
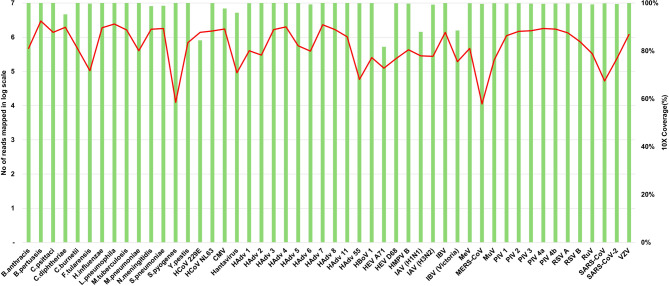
The number of reads mapped in log scale and genome 10X coverage (%) for positive samples. Green bars represent the number of reads mapped on a log scale (left axis), and red lines represent 10X Coverage (right axis).

The analytical performance of the panel was evaluated using 50 target species (36 viruses and 14 bacterial/fungal species) (Fig. 1). The 10× genome coverage for these pathogens ranged from 81.72% to 100%. Mapped read counts varied from 11,152 to 2,966,718 (log₁₀ scale: 4–6). For viral pathogens, human coronavirus 229E (HCoV-229E, 84.42%), human enterovirus A71 (HEV-A71, 81.72%), influenza A virus (IAV, H1N1, 87.95%), and influenza B virus (IBV, Victoria, 88.51%) showed relatively lower coverage. The remaining 32 viral species all achieved ≥ 96% coverage. All 14 bacterial and fungal species achieved ≥ 95% coverage (Fig. 2).

### Limit of detection

**Fig. 3:**
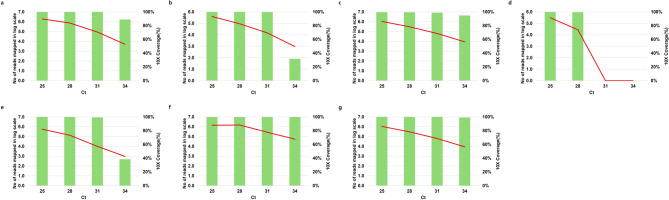
Limit of detection for positive samples. Green bars represent the number of reads mapped on a log scale (left axis), and red lines represent 10X Coverage (right axis). (**a**) Human adenovirus type 4 (HAdV 4), (**b**) mumps orthorubulavirus (MuV), (**c**) Influenza virus A (IAV, H3N2), (**d**) SARS-CoV-2, (**e**) respiratory syncytial virus B (RSV B), (**f**) Parainfluenza virus 2 (PIV 2), (**g**) *Bordetella pertussis* (*B.pertussis*).

To determine the limit of detection (LOD), clinical specimens were serially diluted and analyzed. Notably, mumps orthorubulavirus (MuV) and respiratory syncytial virus B (RSV B) maintained ≥ 80% genome coverage at 10× depth at Ct 31. SARS-CoV-2 reached similar coverage at Ct 28. Influenza A virus (IAV, H3N2), human adenovirus type 4 (HAdV-4), parainfluenza virus 2 (PIV-2), and *Bordetella pertussis* retained ≥ 80% coverage even at Ct 34 (Fig. 3).

### Assessment of capture sequencing in clinical samples

**Fig. 4:**
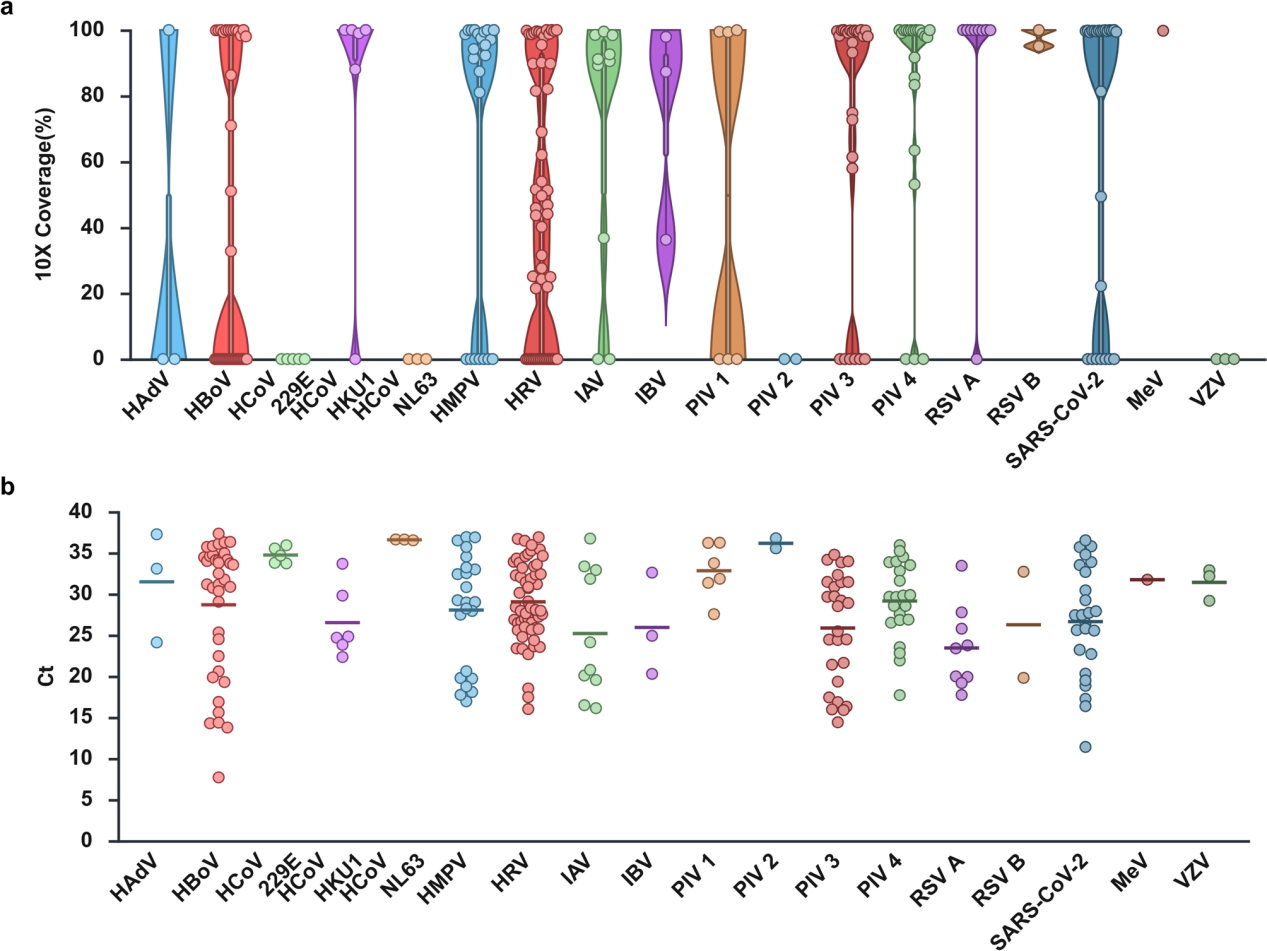
Validation clinical sample for virus. (**a**) Violin plots showing the fraction of viral genomes covered at > 10× depth obtained from NGS data. (**b**) Distribution of Ct values obtained from RT-PCR assays for the corresponding samples. Each dot represents an individual clinical sample. Horizontal lines indicate medians. Created with BioRender.com.

In clinical evaluation, 20 viral species and 5 bacterial species were detected (Table 1). Viral pathogens were identified in 268 samples, of which 154 (57.5%) exhibited ≥ 90% genome coverage at 10× depth (Fig. 4). Among 113 virus-positive samples with Ct ≥ 30, pathogens were not detected in 76 cases by NGS. However, human bocavirus 1 (HBoV-1) and human rhinovirus (HRV) were detected in 1 and 13 samples, respectively, despite being undetectable by multiplex RT-PCR.

**Table 1:** Viral and bacterial pathogens detected by hybrid captured-based NGS.

	#case/Total (%)
Pathogen detected	268/561 (47.77%)
**Virus**	213/561 (37.97%)
Human adenovirus (HAdV)	1/561 (0.18%)
Human bocavirus (HBoV)	18/561 (3.21%)
Human Coronavirus HKU1 (HCoV HKU1)	5/561 (0.89%)
Human metapneumovirus A (HMPV A)	11/561 (1.96%)
Human metapneumovirus B (HMPV B)	5/561 (0.89%)
Human rhinovirus A (HRV A)	30/561 (5.345%)
Human rhinovirus B (HRV B)	10/561 (1.78%)
Human rhinovirus C (HRV C)	10/561 (1.78%)
Influenza virus A (IAV)	8/561 (1.42%)
Influenza virus B (IBV)	3/561 (0.53%)
Parainfluenza virus 1 (PIV 1)	3/561 (0.53%)
Parainfluenza virus 3 (PIV 3)	23/561 (4.10%)
Parainfluenza virus 4 (PIV 4)	19/561 (3.39%)
Respiratory syncytial virus A (RSV A)	8/561 (1.43%)
Respiratory syncytial virus B (RSV B)	2/561 (0.36%)
SARS-CoV-2	18/561 (3.21%)
Measles morbillivirus (MeV)	1/561 (0.178%)
Epstein-Barr virus (EBV)	21/561 (3.74%)
Cytomegalovirus (CMV)	8/561 (1.43%)
Human herpesvirus 6 (HHV-6)	9/561 (1.60%)
**Bacteria**	55/561 (9.80%)
*Bordetella pertussis* (*B. pertussis*)	3/561 (0.53%)
*Mycobacterium avium* (*M. avium*)	1/561 (0.18%)
*Mycobacterium tuberculosis* (*M. tuberculosis*)	1/561 (0.18%)
*Mycoplasma pneumoniae* (*M. pneumoniae*)	40/561 (7.13%)
*Streptococcus pyogenes* (*S. pyogenes*)	10/561 (1.78%)

**Fig. 5:**
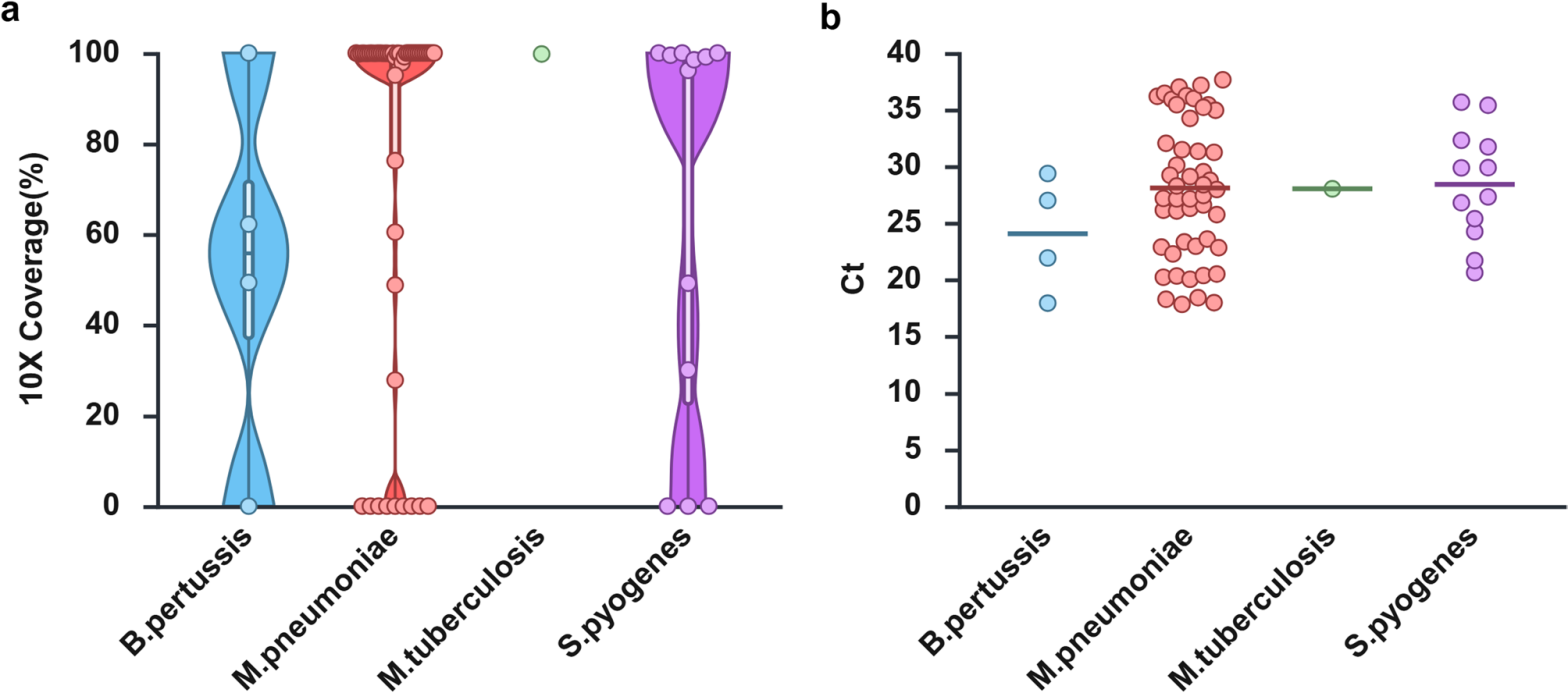
Validation clinical sample for bacteria. (**a**) Violin plots showing the fraction of bacterial genomes covered at > 10× depth obtained from NGS data. (**b**) Distribution of Ct values obtained from RT-PCR assays for the corresponding samples. Each dot represents an individual clinical sample. Horizontal lines indicate medians. Created with BioRender.com.

Bacterial pathogens were identified in 55 samples, with 46 (83.6%) achieving ≥ 90% coverage (Fig. 5). Among 22 bacterial samples with Ct ≥ 30, 11 showed no detectable signal, including 9 *Mycoplasma pneumoniae* (Ct 35.2–37.63) and 2 *Streptococcus pyogenes* (Ct 35.38, 35.65).

**Fig. 6:**
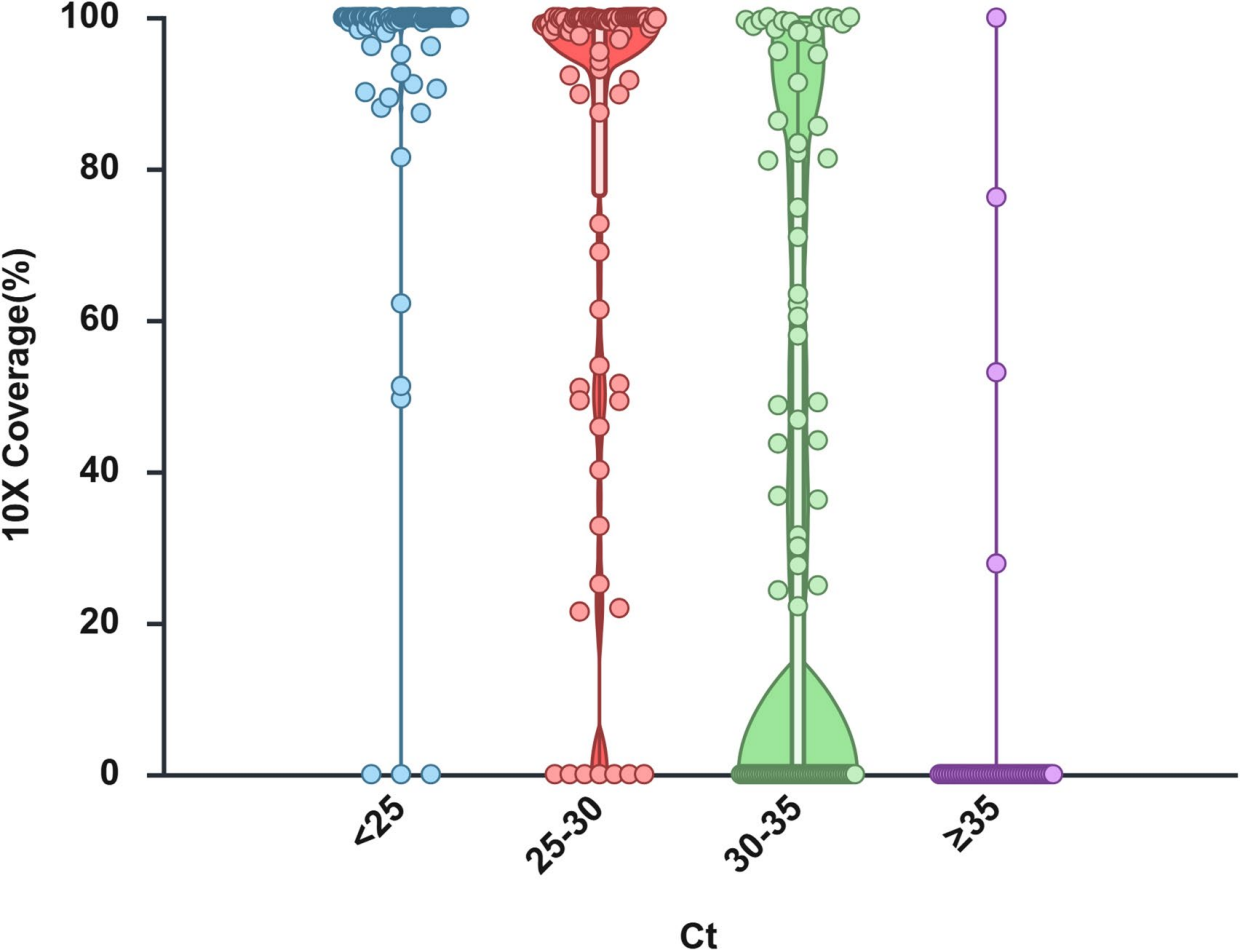
Genome coverage across pathogens according to RT-PCR Ct values. Violin plots showing the distribution of genome coverage (> 10× depth) obtained from NGS data in clinical samples, stratified by RT-PCR cycle threshold (Ct) values. Each dot represents an individual sample measured by RT-PCR. Horizontal lines indicate medians. Created with BioRender.com.

The relationship between Ct values and sequencing coverage is illustrated in Fig. 6. Samples with Ct < 25 consistently achieved > 95% coverage, whereas samples with Ct ≥ 30 often approached 0% coverage. Pathogen-specific results revealed variable coverage reduction, as detailed in Supplementary Figure S1 and Supplementary Table S5.

### Performance relative to multiplex RT-PCR

The performance of the hybrid capture-based NGS panel was evaluated by calculating accuracy, sensitivity, and specificity in comparison with multiplex RT-PCR, which served as the reference standard. High sensitivity and specificity were observed for clinically important pathogens, including influenza A virus (80.0% and 100%), influenza B virus (100% and 100%), and RSV A (88.9% and 100%). Pathogen-specific values for sensitivity, specificity, positive predictive value (PPV), negative predictive value (NPV), and accuracy are presented in Supplementary Table S6.

### Co-infections

**Fig. 7:**
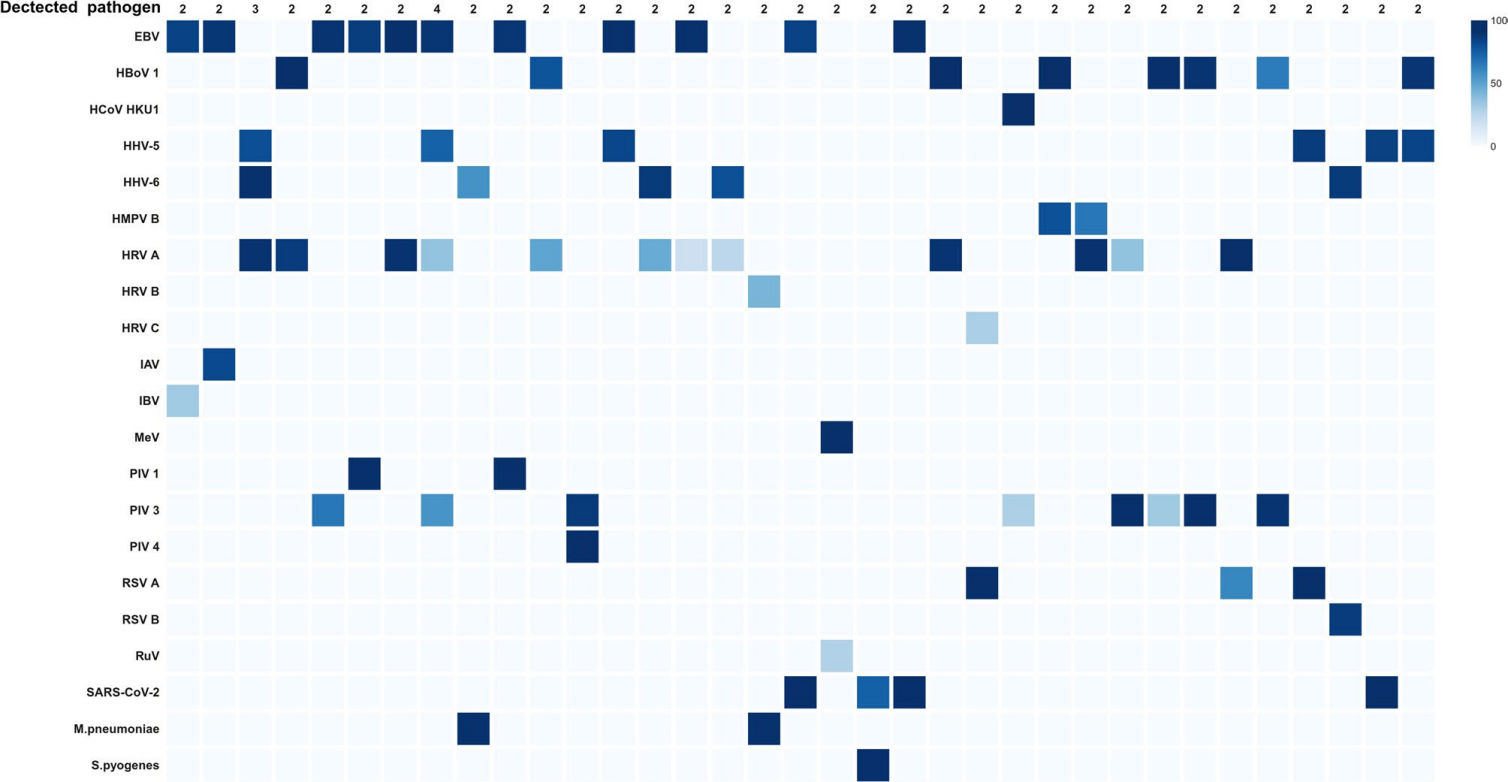
Co-infected pathogen detected in respiratory specimens (*n* = 35) by hybrid-capture sequencing. Heatmap of 10x coverage (%) represented at the species level (created using Bio-render).

Co-infections were identified in 35 samples. Viral–viral co-infections were observed in 32 cases, while 3 cases involved viral–bacterial co-infections. Representative examples included *M. pneumoniae*/HHV-6, *M. pneumoniae*/HRV B, and *S. pyogenes*/SARS-CoV-2 (Fig. 7).

## Discussion

In this study, we developed a hybrid capture-based next-generation sequencing (NGS) panel capable of simultaneously detecting 85 respiratory pathogens (62 viruses and 23 bacterial and fungal species) that are major causative agents of respiratory syndromes. The performance of the panel was evaluated using standardized reference materials and clinical specimens. Among the 50 pathogens tested, genome coverage at 10× depth ranged from 81.72% to 100%, with most viral targets exceeding 96%. A few viruses, including HCoV-229E, HEV-A71, IAV, and IBV, showed relatively lower coverage (81.7–88.5%), likely due to intrinsic genomic features or sequencing-related factors^14^.

Analysis of 561 clinical respiratory specimens demonstrated that most samples achieved ≥ 90% genome coverage at 10× depth, supporting the clinical applicability of the panel. Notably, pathogens such as HBoV-1 and HRV were detected in high-Ct samples (≥ 30), where RT-PCR often fails, underscoring the high analytical sensitivity of the approach. Even under low pathogen load conditions (Ct 28–34), the panel consistently maintained ≥ 80% coverage, confirming its robustness. Importantly, sequencing coverage showed an overall inverse correlation with Ct values, suggesting that the platform can provide semi-quantitative information on pathogen load in addition to qualitative detection. While this inverse relationship was broadly consistent across pathogens, the degree of coverage reduction varied, likely reflecting differences in genome structure, probe accessibility, or sequencing efficiency (Supplementary Fig. S1, Supplementary Table S5). These findings highlight the translational utility of the panel as it complements Ct values with sequencing-based metrics.

The panel also achieved high accuracy, sensitivity, and specificity for major respiratory pathogens when benchmarked against multiplex RT-PCR, the current clinical reference standard. Strong performance was observed for pathogens with sufficient sample sizes, suggesting possible translational relevance. Nevertheless, for pathogens with limited positive cases, wide confidence intervals warrant cautious interpretation; thus, studies should be conducted at a larger scale to validate these findings.

Beyond incremental detection, the clinical utility of the panel lies in its ability to complement RT-PCR by detecting pathogens in high-Ct samples, comprehensively profiling co-infections, and capturing novel variant or emerging pathogens^5,15,]^^16^. These advantages may be particularly valuable in unexplained severe respiratory infections, in immunocompromised patients, and in the context of public health surveillance for novel pathogens.

Co-infection analysis revealed 35 cases, including 32 viral–viral and 3 viral–bacterial infections. These results indicate that the panel can effectively identify not only single infections but also complex co-infection scenarios. The platform also enables direct whole-genome sequencing without the need for culture or PCR amplification, and the use of probes designed from diverse reference genomes enhances the detection of genomic variants and pathogen diversity.

Unlike most commercially available respiratory panels, which typically focus on either viruses or bacteria, the panel described here can simultaneously detect viruses, bacteria, and fungi on a single platform, demonstrating broad applicability and scalability^8,9^. Nonetheless, reduced coverage or detection failure observed for certain pathogens may be addressed by refining probe design and optimizing sample preprocessing. Integration into real-time infectious disease surveillance could further enhance early detection and strengthen public health responses.

This study has several limitations. First, because probe design was based on reference sequences, the ability to detect novel pathogens is limited. This can be complemented by non-targeted approaches such as metagenomic NGS^17^. Second, sensitivity and specificity may be influenced by factors such as sample preprocessing, library quality, and sequencing platform performance, emphasizing the need for rigorous standardization. Third, optimization of nucleic acid extraction protocols is required to improve detection of bacterial and fungal pathogens. Additionally, validation across diverse specimen types, including blood, urine, and stool, should be pursued in future studies^18,19^.

In conclusion, the hybrid capture-based NGS panel developed here is a robust and effective tool for high-sensitivity detection of diverse respiratory pathogens. It has potential for application in public health systems, such as those operated by the Korea Disease Control and Prevention Agency, to support early identification of pathogens of unknown etiology, infectious disease surveillance, and rapid response. Integration with machine learning–based analytics and automated reporting could further evolve this platform into a faster and more precise diagnostic support tool.

## Methods

### Panel design

For the selected pathogens, the target regions were determined based on well-established markers in conventional assays. For the viral panel, the entire genomes of all viral pathogens were designated as capture regions. For the bacterial and fungal panels, specific genes commonly employed for pathogen differentiation were selected as targets, and the surrounding regions were included to ensure sufficient capture efficiency. The panel was specifically designed to detect pathogens at the species level rather than the strain level. Subsequently, in silico analyses were performed to exclude sequences with high similarity to other microorganisms, resulting in the identification of the final capture regions. Probes were then designed and synthesized using a 2× tiling strategy across these regions.

### In-silico analysis

Using reference genomes and the initial capture region for the targeted pathogens, virtual DNA fragments mimicking the target-captured sequences were generated using an in-house program. Subsequently, virtual Next-Generation Sequencing (NGS) data incorporating a 10% mutation ratio for these fragments were produced using the wgsim (v. 0.3.1-r13) program with the following parameters: “-h -d 200 -s 40 -r 0.01”^20^. The simulated NGS data were then assembled de novo using Megahit assembler (v. 1.2.9) with the following parameters: “--k-min 121 –k-max 141 –k-step 10 –min-contig-len 800 –min-count –prune-level 3 –no-mercy” ^21^. The resulting contigs were aligned using BLAST (v. 2.14.1+) with default parameters against both the reference genomes of the target pathogens and multiple available strain genomes^22^. The reference genome with the highest similarity was consistently identified as the intended target species, thereby confirming that the designed panel enables detection at the species level without cross-reactivity across different species.

### Clinical sample and positive control

We evaluated a total of 611 samples, including 50 positive controls and 561 patient samples. Positive controls consisted of clinical specimens, cultured isolates, synthetic constructs, and commercial reference materials (Vircell, Spain). The origin and composition of positive controls, including type, source, and concentration are presented in Supplementary Table S7. These controls were primarily used for assay validation and limit of detection (LoD) testing. Seven positive controls were serially diluted to Ct values of 25, 28, 31, and 34 to evaluate the analytical sensitivity. Clinical samples comprised 561 patient-derived specimens, which were tested using PowerChek™ 36 Respiratory Pathogen Panel (KOGENEBIOTECH, South Korea). Of these, 506 were nasal swabs, 50 were sputum, and 5 were bronchoalveolar lavage fluid (BALF) samples, yielding 204 positive and 357 negative results for syndromic respiratory virus/bacterial multiplex RT-PCR. The temporal distribution of sample collection ranged from October 2023 to December 2024. Nucleic acid (NA) was extracted from clinical samples using the QIAsymphony DSP Virus/Pathogen Mini Kit (Qiagen, Germany) on a QIAsymphony SP instrument, with 280 µl of input volume and 110 µl of elution.

### Target capture and sequencing

A Celemics Double-Stranded cDNA Synthesis Kit (Celemics, South Korea) was used to generate cDNA from nucleic acids extracted from each pathogen sample. For the first-strand synthesis, the RNA sample was mixed with a primer and dNTP mixture, and incubated at 65 °C for 5 min. After the incubation, the mixture was cooled on ice. Then, first Strand Synthesis Buffer-1, 1st Strand Synthesis Buffer-2, RNase inhibitor, and reverse transcriptase were added, followed by incubation in the thermal cycler with the following program: 23 °C for 10 min, 50 °C for 10 min, 80 °C for 10 min, and cooling at 4 °C. For the 2nd strand synthesis, second Strand Synthesis Buffer-1, 2nd Strand Synthesis Enzyme-1 and 2nd Strand Synthesis Enzyme-2 were mixed with the first Strand cDNA product and incubated at 16 °C for 60 min, followed by cooling at 4 °C. Then, 2nd Strand Synthesis Buffer-2 and second Strand Synthesis Enzyme-3 were added and incubated at 25 °C for 15 min, followed by cooling at 4 °C. After running the thermal cycler program, second-strand synthesis clean-up was performed with CeleMag Clean-up Beads.

Subsequently, the Celemics EP Kit was used to construct an NGS library. EP-ER/A Buffer and EP-ER/A Enzyme were mixed with synthesized cDNA and incubated in the thermal cycler with the following program: 4 °C for 1 min, 32 °C for 18 min, 65 °C for 30 min, and cooling at 4 °C. Subsequently, NGS adapter, Ligation Buffer, and Ligation Enzyme were added and incubated at 20 °C for 20 min. The prepared library was purified using CeleMag clean-up beads. After purification, the NGS library was amplified with CLM Polymerase and Celemics UDI (Unique Dual Index) primers for 8–12 PCR cycles, depending on the initial sample amount. The amplified library was purified using CeleMag clean-up beads.

Target enrichment was performed using the synthesized pathogen panel and the Celemics Target Enrichment Kit. For target enrichment, Block #1, Block #2, and Block #3 were mixed with the NGS library sample and incubated at 95 °C for 5 min, followed by holding at 65 °C. The hybridization Buffer and Enhancer mixture were then incubated at 65 °C for 3 min. Subsequently, Target Capture Probe #1, designed for capturing viral or bacterial/fungal target sequences, and Target Capture Probe #2, an auxiliary mix formulated to improve hybridization efficiency, were combined and incubated at 65 °C for 2 min. After incubation, the hybridization buffer mixture was added to the Target Capture Probe mixture, and the sample-blocker mixture was transferred to the probe-buffer mixture. The final mixture was incubated at 65 °C overnight for hybridization capture. The Target Captured Library was selected using CeleMag Streptavidin Beads and recovered by post-capture amplification using CLM polymerase and Post Capture PCR primers. The amplified library was purified using CeleMag clean-up beads. Sequencing was performed using an Illumina NextSeq 500 or NextSeq 2000 platform. No-template controls (NTCs) were processed in parallel throughout extraction, library preparation, target enrichment, and sequencing, and consistently yielded no detectable target reads.

### NGS data analysis

The generated NGS data underwent quality filtering using an in-house program and the AdapterRemoval (v. 2.3.1) with the following parameters: “--trimns –trimqualities –minquality 20 –minlength 30”^23^, eliminating bases with low quality or adapter content. Subsequently, alignment to the human reference genome was performed using the BWA algorithm (v.0.7.17.r1198) with default parameters to remove sequences originating from the human genome^24^. The refined reads were then assembled into contigs using Megahit assembler (v. 1.2.9) with the following parameters: “--k-min 121 –k-max 141 –k-step 10 –min-contig-len 800 –min-count –prune-level 3 –no-mercy”^21^. Contigs with a supporting read depth of fewer than 30 were excluded to minimize low-level background signals, including potential index crosstalk. The contigs were aligned to the reference genomes of each pathogen using the Basic Local Alignment Search Tool (BLAST, v 2.14.1+) with default parameters^22^. Based on the BLAST results, candidate pathogen genomes were selected by considering parameters such as the Percentage of Identity (PID), alignment length, query coverage, and E-value using an in-house program. The refined reads were then realigned to the selected pathogen genomes using BWA alpha with default parameters. PCR duplicates were removed using Picard Mark Duplicates software (v. 2.27.2)^25^. The coverage information for the target regions of the panel was assessed using an in-house program. Pathogens with ≥ 20% of the 10X coverage were considered positive detections, whereas those with ≥ 10% but < 20% of the 10X coverage were regarded as potential detections.

### Statistical analysis

Correlations between RT-PCR cycle threshold (Ct) values and sequencing coverage were assessed using Spearman’s rank correlation. Data distributions were visualized as violin plots, which were generated using BioRender.

Sensitivity, specificity, PPV, NPV, and accuracy were calculated using 2 × 2 contingency tables, with multiplex RT-PCR serving as the reference standard. 95% confidence intervals (95% CIs) were estimated using the Clopper–Pearson method. A two-sided p value of < 0.05 was considered statistically significant.

## Supplementary Information

Below is the link to the electronic supplementary material.

Supplementary Material 1

Supplementary Material 2

Supplementary Material 3

## Data Availability

The sequencing data generated in this study have been deposited in the NCBI Sequence Read Archive (SRA) under the BioProject accession number PRJNA1336226.
