# Supplementary material for: Development and evaluation of a hybrid capture-based NGS panel for comprehensive detection of respiratory pathogens
Source: Sci Rep. 2025 Nov 26;15:42238. doi: 10.1038/s41598-025-26421-2 (PMC12658075; doi:10.1038/s41598-025-26421-2)
Supplement: Supplementary file 1 — Supplementary Material 1 [file 41598_2025_26421_MOESM1_ESM.pdf]

Development and Evaluation of a Hybrid Capture-Based NGS Panel for Comprehensive Detection of Respiratory Pathogens

Jeong-Ah Kim[1], Jeong-Min Kim[1], Chaeyoung Lee[1], Il-Hwan Kim[1], Daehwan Lee[2], Heesoo Lee[2], Jaehwan Jeong[2], Eun-Jin Kim[1]*

[1] Division of Emerging Infectious Diseases, Department of Laboratory Diagnosis and Analysis, Korea Disease Control and Prevention Agency (KDCA), Cheongju, Republic of Korea

[2] R&D team, Celemics, Inc., Seoul, Republic of Korea

***Corresponding author**

Eun-Jin Kim, Ph.D.

Division of Emerging Infectious Diseases, Department of Laboratory Diagnosis and Analysis, Korea Disease Control and Prevention Agency (KDCA), Osong Health Technology Administration Complex, 187, Osongsaengmyeong 2-ro,

Osong-eup, Heungdeok-gu, Cheongju-si, Chungcheongbuk-do, Korea

E-mail: ekim@korea.kr

Tel +82 043-719-8140, Fax +82 043-719-8229

Supplementary Figure S1. Correlation between RT-PCR Ct values and genome coverage in individual pathogens

Violin plots illustrating the distribution of genome coverage (>10× depth) obtained from NGS data in relation to RT-PCR Ct values for individual pathogens. Only pathogens with ≥5 clinical samples are displayed. Each dot represents an individual sample. Horizontal lines represent medians, as in Figure 6. Viral pathogens are shown in panel a, and bacterial pathogens are depicted in panel b. Created with BioRender.com.
