# Supplementary figures and images for: Development and evaluation of a hybrid capture-based NGS panel for comprehensive detection of respiratory pathogens
Source: Sci Rep. 2025 Nov 26;15:42238. doi: 10.1038/s41598-025-26421-2 (PMC12658075; doi:10.1038/s41598-025-26421-2)

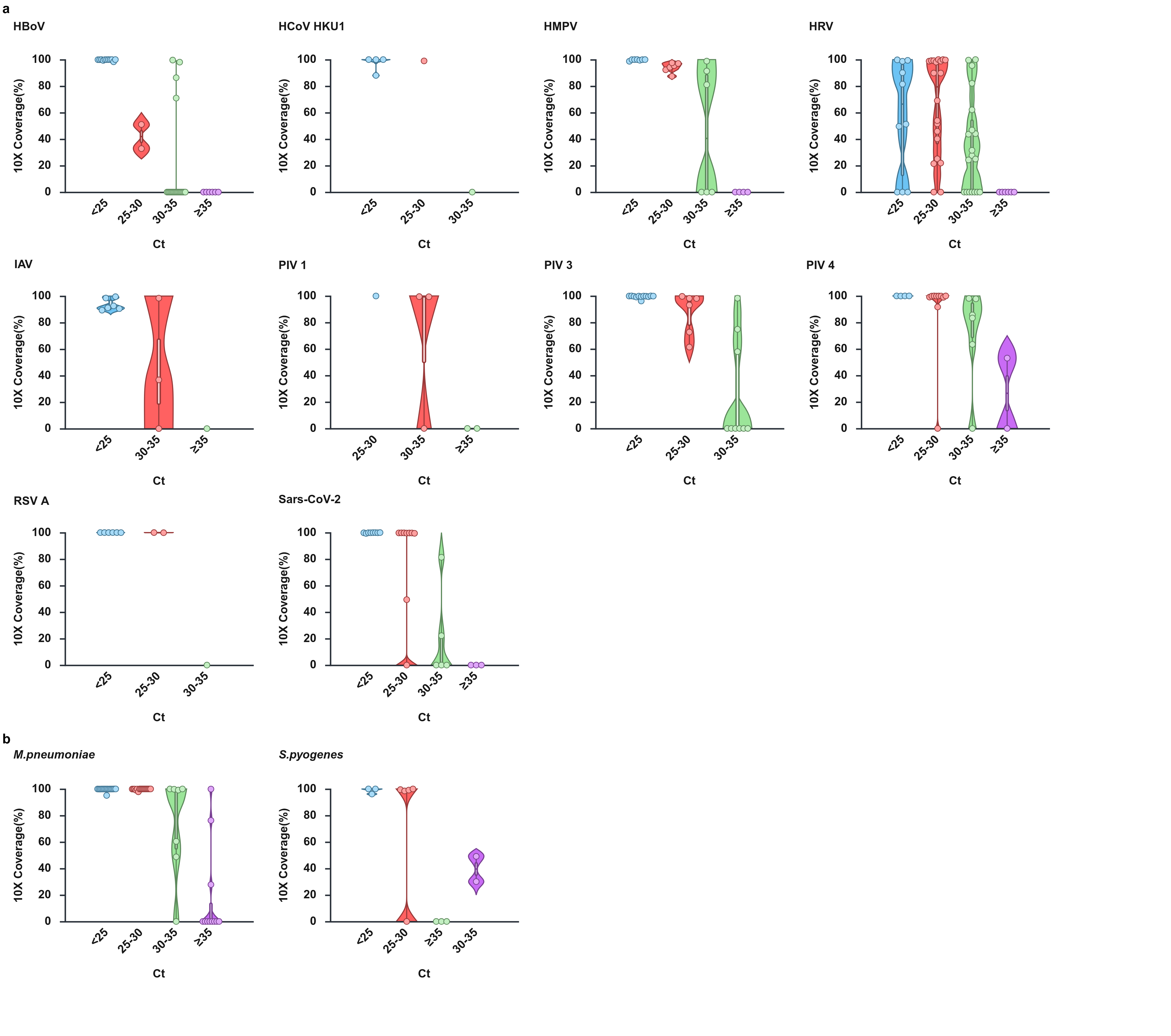

Supplement: Supplementary file 2 — Supplementary Material 2 [file 41598_2025_26421_MOESM2_ESM.png]
